# A Deep Learning Approach for Detecting Colorectal Cancer via Raman Spectra

**DOI:** 10.34133/2022/9872028

**Published:** 2022-04-07

**Authors:** Zheng Cao, Xiang Pan, Hongyun Yu, Shiyuan Hua, Da Wang, Danny Z. Chen, Min Zhou, Jian Wu

**Affiliations:** ^1^RealDoctor AI Research Center, College of Computer Science and Technology, Zhejiang University, China; ^2^Key Laboratory of Cancer Prevention and Intervention, Ministry of Education, The Second Affiliated Hospital, Zhejiang University School of Medicine, China; ^3^Institute of Translational Medicine and the Cancer Institute of the Second Affiliated Hospital, Zhejiang University School of Medicine, China; ^4^Department of Computer Science and Engineering, University of Notre Dame, USA; ^5^Second Affiliated Hospital School of Medicine, and School of Public Health, Zhejiang University, China

## Abstract

*Objective and Impact Statement.* Distinguishing tumors from normal tissues is vital in the intraoperative diagnosis and pathological examination. In this work, we propose to utilize Raman spectroscopy as a novel modality in surgery to detect colorectal cancer tissues. *Introduction.* Raman spectra can reflect the substance components of the target tissues. However, the feature peak is slight and hard to detect due to environmental noise. Collecting a high-quality Raman spectroscopy dataset and developing effective deep learning detection methods are possibly viable approaches. *Methods.* First, we collect a large Raman spectroscopy dataset from 26 colorectal cancer patients with the Raman shift ranging from 385 to 1545 cm${\;}^{-1}$. Second, a one-dimensional residual convolutional neural network (1D-ResNet) architecture is designed to classify the tumor tissues of colorectal cancer. Third, we visualize and interpret the fingerprint peaks found by our deep learning model. *Results.* Experimental results show that our deep learning method achieves 98.5% accuracy in the detection of colorectal cancer and outperforms traditional methods. *Conclusion.* Overall, Raman spectra are a novel modality for clinical detection of colorectal cancer. Our proposed ensemble 1D-ResNet could effectively classify the Raman spectra obtained from colorectal tumor tissues or normal tissues.

## 1. Introduction

Colorectal cancer (CRC) is a common health issue, with an estimated 148,000 new cases and 53,000 deaths in America in 2020 [1]. In order to reduce the incidence and mortality of colorectal cancer, the effectiveness of guaiac fecal occult blood test (gFOBT) and sigmoidoscopy screening has been studied in randomized controlled trials [2–11]. Colonoscopy is likely to reach the entire large intestine at least as effectively as sigmoidoscopy, which reaches only the far end of the large intestine [12]. Colonoscopy is a primary test for diagnosing colorectal cancer. This process also has the potential to prevent diseases by eliminating precancerous lesions and thus is an important tool to help improve clinical outcomes. Unfortunately, the colonoscopy test is not 100% accurate, and cancer can appear months or years later when a colonoscopy test is negative for cancer. The World Endoscopy Organization (WEO) defines such cases as postcolonoscopy of colorectal cancer (PCCRCs) [13]. There is evidence that as many as 700 patients in the National Health Service (NHS) are diagnosed with PCCRCs each year [14]. Therefore, improving the detection rate of colorectal cancer is vital.

Raman spectroscopy is a nondestructive chemical analysis technique that attains the spectral characteristics of tissues based on the molecular characteristics generated by the inelastic scattering of the incident light. Inelastic scattering occurs when light interacts with matter, but its relative importance is reduced by competing phenomena, including elastic scattering and absorption [15]. Raman spectroscopy is used to observe low-frequency vibration patterns in the system. Raman spectral results provide a fingerprint through which different molecular species can be identified and their relative concentrations assessed according to the intensities of different peaks. Biological tissues, such as intestinal tissue, contain a large number of Raman active molecules, resulting in a spectral measurement that is actually a weighted spectral sum from all the molecular species contained in the target tissue volume [16].

At present, colonoscopy is based on biopsy or endoscopic tissue characteristics and in vivo classification. Color endoscopy and Kudo classification are the main auxiliary examinations for colon lesions [17], but it is difficult to identify some small lesions from the normal intestinal mucosa. Therefore, clinical applications genuinely need a noninvasive, rapid, and high-precision diagnostic tool to detect some early curable colorectal cancer. In addition, early detection requires better clinical instruments than colonoscopy biopsies, which can be extended to a wider population rather than limited by time and costs. In order to help address this critical problem, Raman spectroscopy, a new diagnostic tool featuring high speed, data preservation, and fine accuracy, has been verified in many comprehensive studies, making it possible to be applied in future clinical practice [18–22]. By comparing the Raman spectra of tumor tissues with those of normal tissues, it is possible to find the main Raman spectrum characteristics that reflect these different tissues.

Recently, numerous machine learning methods have been applied in spectral analyses [23]. Principle component analysis (PCA) is the most commonly used one, which extracts the top variances that contribute to the comprehensive information. Some multivariate classifiers such as linear discriminant analysis (LDA) or clustering methods (e.g., k-nearest neighbor (KNN)) can be applied to further distinguish the targets through the PCA results [24]. Support vector machines (SVM) is another powerful tool, which enables linear classification of the target data in a higher dimension space by determining a hyperplane [25, 26]. Partial least square regression (PLSR) is a bilinear factor method that allows relating two data matrices through a linear multivariate model [27]. Artificial neural networks (ANNs) can separate data categories by passing information through successive neuron layers [28]. However, these methods can hardly learn more in-depth information within the spectra data, such as the wavelength differences of subtle peaks, because they consider only the disperse input points while neglecting the internal relations.

As a subset of machine learning methods, deep learning is a promising technique to extract effective features across multiple levels of abstraction, which has demonstrated state-of-the-art performance in a large number of challenging tasks such as medical image recognition [29–32]. Deep learning applications on Raman spectroscopy data also achieved promising results in various tasks such as Raman denoising [33], brain tumor detection [34], and pathogenic bacteria identification [35]. Compared with other machine learning methods, deep learning models can capture information from shared convolution kernels without manually selecting features. Among the above applications, one-dimensional convolutional neural networks (1D-CNNs) are effective deep learning models and have been widely used in spectra recognition [36]. However, information transmission loss in CNNs may cause gradient loss and damage the model performance. Inspired by the success of residual connected networks against gradient loss, a one-dimensional residual convolutional neural network (1D-ResNet) has been utilized to improve the performance of spectra classification [37].

Focusing on tumor detection, Ralbovsky and Lednev reviewed machine learning methods in medical diagnosis applications with Raman spectroscopy [23]. Based on spontaneous Raman spectroscopy (RS) and surface-enhanced Raman spectroscopy (SERS), a large set of studies analyzed cancer data with machine learning methods, and the types of cancer examined included brain, breast, cervix, and liver. On colorectal cancer, Gala de Pablo et al. investigated five different colorectal cell lines with spontaneous RS and utilized a PCA-LDA method to obtain a 92.4% classification accuracy [38]. Králová used SERS to analyze blood plasma differences between normal persons and oncological patients [39]. Still, most known RS studies on cancer diagnoses relied on traditional machine learning methods like PCA to extract features and classify sample types, but, due to limited dataset sizes, the results of the previous studies are not general and comprehensive enough.

In this work, we aimed to develop a new colorectal cancer detection method with Raman spectroscopy. For this purpose, the effects of different tissues on Raman spectra were investigated. Furthermore, we designed a deep learning architecture to classify tumor tissues through their Raman spectra. Comparison experiments and visualization results demonstrated that our deep learning approach can detect colorectal cancer fast and accurately. Our work could make it possible for Raman spectroscopy combined with colonoscopy to improve the detection rate of colorectal cancer in the future.

## 2. Materials and Methods

### 2.1. Study Design

**Figure 1:**
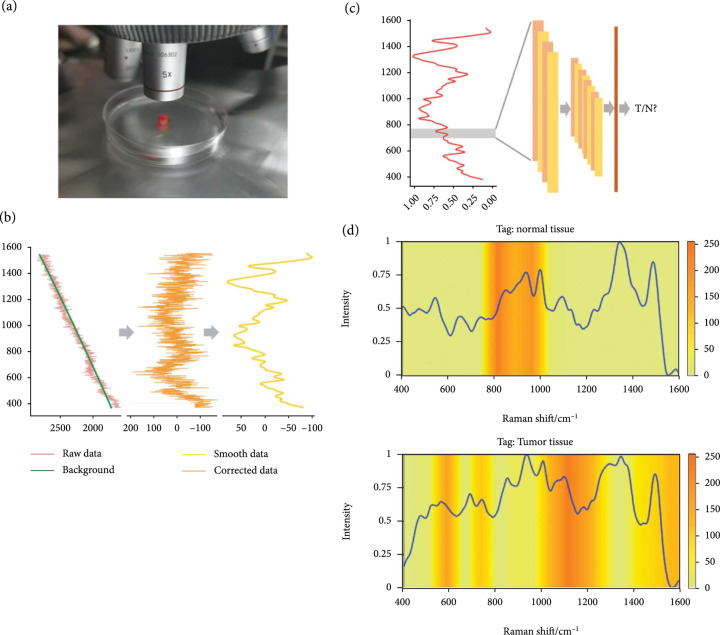
A schematic diagram of our proposed deep learning approach for detecting colorectal cancer. (a) Preparing tissues and scanning with a Raman spectrometer using ×20 microscope objective lens. (b) Preprocessing the original data, including baseline correction, denoising, and normalization. (c) Training the one-dimensional residual convolutional neural network to classify two tissue types from Raman spectral data. (d) Visualizing the neural network by Grad-CAM and interpreting the meaning of activated peaks with deep learning.

The objective of this study was to evaluate the potential of using Raman spectroscopy to distinguish colorectal cancer from normal tissues in grades 1 to 3 adenocarcinoma. This study investigated the capability of Raman spectroscopy for intraoperative use on adult patients of colorectal cancer surgery at the Second Affiliated Hospital of Zhejiang University School of Medicine, China with grades 1 to 3 adenocarcinoma. All the patients included in this study gave written informed consent and were fully aware of the aims of the study. The surgeons were blinded to any information about the acquired Raman spectra during the resection procedures. The pathologists were blinded to any information about the Raman spectra before performing histological analyses. An overall schematic diagram of our proposed approach is shown in Figure 1.

### 2.2. Sample Preparation

**Figure 2:**
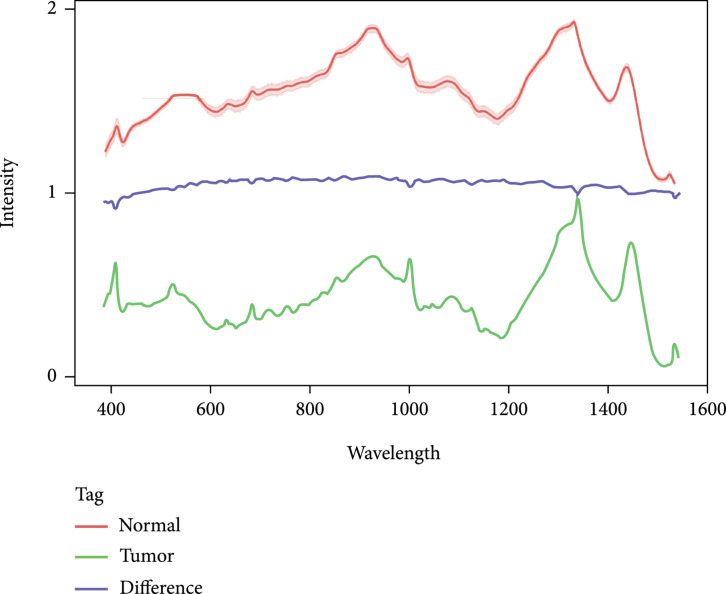
An example possibility plot of the collected Raman spectra of CRC and normal tissues, normalized to [0, 1]. The upper red curve represents CRCs, and the below green curve represents normal tissues, with only slight differences (blue curve) between the two sample types.

This study has been approved by the Second Affiliated Hospital of Zhejiang University School of Medicine and the Institute of Translational Medicine of Zhejiang University. All the tumor tissue samples and paired normal tissues were obtained by surgical resection and stored at −80°C from 2018 at the Second Affiliated Hospital of Zhejiang University School of Medicine. The disease stage was determined based on the pathological tumor-node-metastasis (pTNM) classification system [40]. In summary, there were 26 colorectal cancer samples including 6 grade I, 12 grade II, and 8 grade III. The details were presented in Table 1. All the samples were detected by Raman spectroscopy under tinfoil without any treatment. The results of Raman spectra are shown in Figure 2.

**Table 1:** Description of the collected CRC samples.

	N (%)
Age, years (median, range)	26 (50-73)
Gender	
Male	16 (62%)
Female	10 (38%)
Primary location of neoplasm	
Right colon	10 (39%)
Left colon	8 (31%)
Sigmoid colon	4 (15%)
Rectum	4 (15%)
TNM	
I	6 (23%)
II	12 (46%)
III	8 (31%)
IV	0 (0%)
Grade	
Well differentiated	6 (23%)
Moderately differentiated	18 (69%)
Poorly differentiated	2 (8%)

### 2.3. Raman Spectral Data Acquisition

The Raman spectra were collected in a dark room at $20^{{}^{\circ}}$ with Renishaw in via Raman spectrometer (UK) equipped with the ×20 microscope objective lens. The scanning range was from 385 to 1545 cm^-1^. Each sample was detected three times to obtain an average value. Before each experiment, the Raman spectrometer was calibrated using the 520.5 cm^-1^ bands of a silicon wafer. We split the collected tumor and paired normal tissues into over 20000 small pieces on average and collected the corresponding Raman spectrum of each piece. Afterward, we built the Raman spectrum dataset of CRC, containing 20424 Raman spectrum data. The training set, validation set, and test set account for 80%, 10%, and 10%, respectively. The detail of the CRC Raman dataset is shown in Table 2.

**Table 2:** Description of the collected CRC Raman dataset amount.

Tissue type	Training set#	Validation set#	Test set#	Overall
Normal	8684	1091	1091	10866
Tumor	7654	952	952	9558

### 2.4. Data Preprocessing and Data Augmentation

In the model pretraining stage, we carry out two procedures, for data preprocessing and data augmentation. First, we clean the collected original Raman spectra data to reduce the adverse effects of noise and improve the stability of the mathematical model. As illustrated in Figure 1(b), there are mainly three steps:

(i) Baseline correction: due to the fluorescence effect, there are usually specific peak shiftings that may cause overfitting of the model. The polynomial baselines are fitted by an asymmetric least square algorithm and removed from the original data
(ii) Signal denoising: after the baseline correction, we perform denoising of the Raman spectra data to obtain purer data for analysis
(iii) Data normalization: the final step is scaling, in which the intensity values of all spectra are normalized to the range of [0, 1] so that the minimum intensity value of each spectrum is 0, and the maximum is 1

The data preprocessing procedure is conducted using Python 3.8 and the RamPy package. An example for tumor tissue and normal tissue after preprocessing is given in Figure 2 for comparison, in which the CRC tumor tissue and normal tissue show only slight differences on some shift peaks.

Next, in the data augmentation procedure, we seek to extend our dataset with three operations. First, we generate white Gaussian noise proportional to the magnitude at each Raman shift. Second, we shift each spectrum left or right by a few Raman shifts randomly. Third, we multiply the raw spectra by a random intensity enhancement factor ranging from 0.2 to 2.

### 2.5. Deep Learning Architecture

Convolutional neural networks (CNNs) and their variants such as residual networks (ResNets) have shown very good potential to extract key features from complex data and have been widely successful across a set of image recognition tasks. ResNet uses short connections between the input and output of each residual block to prevent vanishing gradient and overfitting while training a deep network architecture [37].

**Figure 3:**
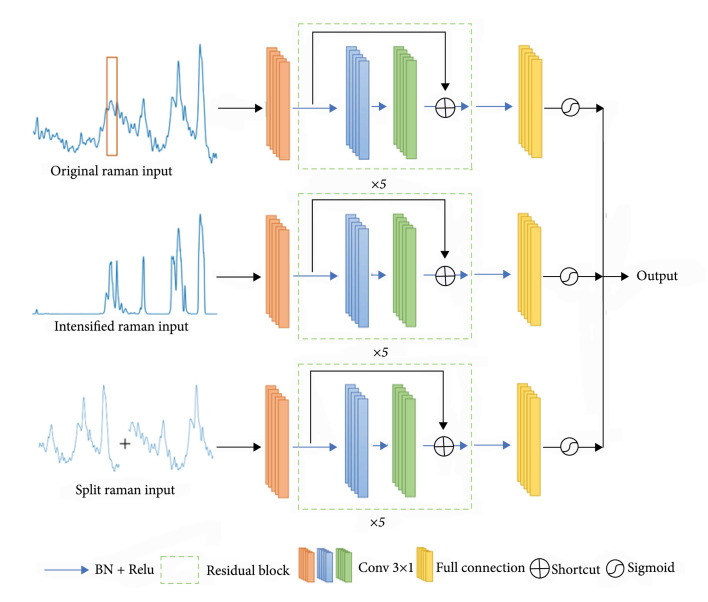
The overall architecture of our proposed ensemble 1D-ResNet model.

We utilize two types of residual bottlenecks, i.e., normal bottleneck and downsample bottleneck, as the blue and green block shown in Figure 3. The input size is defined as $N*C_{\text{in}}*H$, where N donates the batch size, $C_{\text{in}}$ donates the number of input channels, and H donates the height of a feature map. Each residual block contains two batch normalization (BN), activation layer (ReLU), dropout, and convolutional layer sets and has a short connection between input and output. The batch normalization can prevent overfitting effectively, which can be expressed as

(1)BNxk=γxk−μBσB2+ε+β,

where ε donates a random noise, $\mu_{B}$ and $\sigma_{B}$ donate the mean and variance values in the batch, respectively, and γ and β are adaptable parameters in training. In an activation layer, we use rectified linear units (ReLU) as the activation function, which is described as

(2)ReLUx=max0,x.

In a convolutional layer, we set the convolutional kernel size as 1×3. A convolutional layer can be defined as

(3)yj=fbj+∑iwij∗xi,

where $x^{i}$ and $y^{j}$ are the $i^{\text{th}}$ input map and $j^{\text{th}}$ output map, respectively, $w^{ij}$ donates the weight between the extracted features and output, and $b_{j}$ stands for the bias of the $j^{\text{th}}$ map. The downsample bottleneck output size is N∗C∗H/2, while the normal bottleneck output size is N∗C∗H in a convolutional layer.

Each bottleneck contains a shortcut between the input and output feature maps, which can be written as

(4)HX=FX,wi+X,

where X and $H\left(X\right)$ are the input and output vectors of the bottleneck, and $F\left(X,\left\{w_{i}\right\}\right)$ donates the residual mapping to be learned.

Figure 3 illustrates the overall architecture of our proposed 1D-ResNet model used in this work. Since the input Raman spectral data is preprocessed as a 1×1024 vector, the input data size is N∗1∗1024. The 1D-ResNet architecture is similar to ResNet-34 [37]. Apart from the first bottleneck, downsample is conducted after two bottlenecks, until we obtain the feature map of size N∗1024∗1. After batch normalization and activation of the final bottleneck, we use a full connection layer and apply a sigmoid function to calculate the probability, which can be expressed as

(5)Sigmoidx=11+e−x.

### 2.6. Methods in Comparison

The CRC diagnosis using Raman spectra data can be formulated as a binary classification task. To verify the performance of our 1D-ResNet for this task, we conduct comparison experiments using several commonly used machine learning methods, such as SVM, LightBoost, XGBoost, and Random Forest [41]. SVM is a classic linear classifier, XGBoost, and LightBoost are typical boosting methods, and Random Forest is a kind of decision tree method. We perform fine-tuning of the parameters for each of these methods and record its best performance in the validation set.

### 2.7. Implementation Details

The 1D-ResNet is trained with the adaptive moment estimation (Adam) algorithm as the optimizer, which is a variant of the stochastic gradient descent method, for 200 epochs with a learning rate of 0.0001. The batch size is set to 128. To minimize the cost, we use the weighted loss function as defined in Eq. (6):

(6)I=∑i=1nyilnyi^+yi^−yiln1−yi^,

where $y_{i}$ is the label of the $i^{\text{th}}$ spectrum with value 1 or 0, $\hat{y_{i}}$ is the predicted probability of the $i^{\text{th}}$ output by the model, and n is the total sample size.

**Figure 4:**
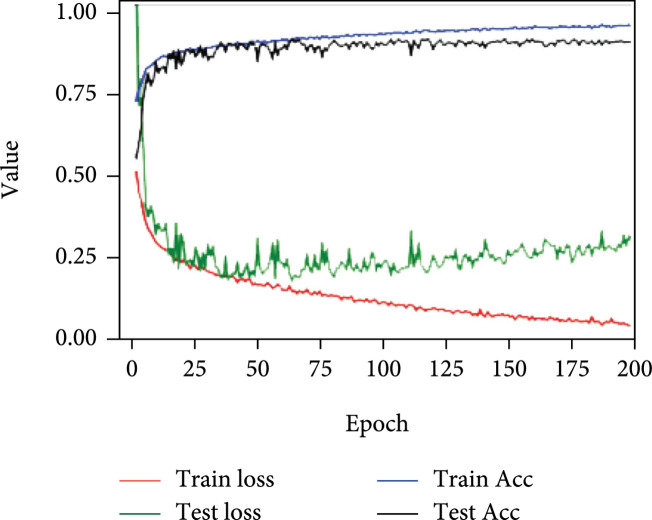
Accuracy and loss curves of the training set and test set with 200 epochs of training.

Figure 4 shows the changes in the accuracy and loss curves of both the training set and test set with various numbers of epochs. As the number of epochs increases, the accuracy tends to increase, while the loss shows a decreasing trend. The accuracy and loss reach stable values after hundreds of epochs of training. We record the best performance when the test accuracy reaches the maximum value.

Our experiments were performed on a workstation with Intel(R) Xeon(R) CPU E5-2630 v4 @ 2.20GHz, 256 GB RAM, and 8× NVIDIA Titan Xp GPU with 12 GB GPU memory. The code was implemented with PyTorch 1.6.1 in Ubuntu 18.04.

## 3. Experiments and Results

### 3.1. Evaluation Metrics

To evaluate the model performance, we report accuracy, precision, recall, and F1-score of each method. The definitions of the evaluation metrics are defined as follows:

(7)Accuracy=TP+TNTP+FP+TN+FN,(8)Recall=TPTP+FN,(9)Precision=TPTP+FP,(10)F1‐score=2×TP2×TP+FN+FP,

where TP, FP, TN, and FN represent the true positive, false positive, true negative, and false negative prediction results, respectively. Furthermore, we report the area under the ROC curve (AUC) while comparing the performance of different machine learning methods.

(11)AUC=12∑i=1m−1TPRi+1+TPRiFPRi+1−FPRi,

where TPR and FPR donate the true positive rate and false positive rate, respectively.

### 3.2. Training Results of 1D-ResNet

**Figure 5:**
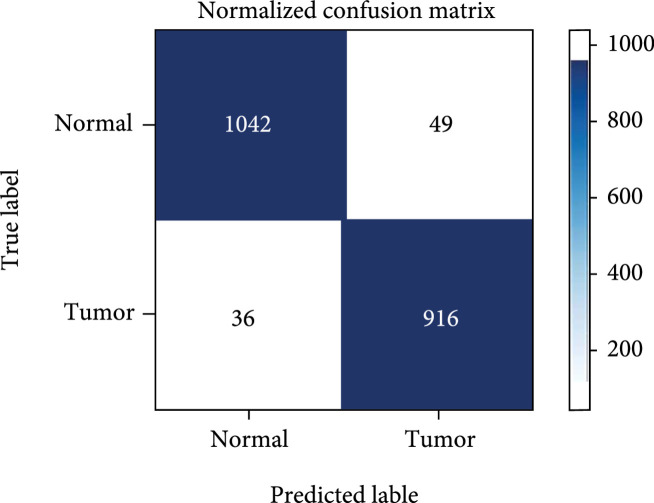
The confusion matrix of our ensemble 1D-ResNet model on the test set.

We train the 1D-ResNet model described in Section 2.5. The accuracy of the model is 94.6%. To enhance the model performance, we utilize three strategies to improve the model. On one hand, we intensify the Raman shift peaks by multiplying some intensity factors with the main peaks in the raw spectra, which is similar to the data augmentation procedure. The accuracy of ResNet_intensify raises to 95.3%. On the other hand, we split the raw data into [0, 512] and [128-640] to capture partial features, especially the parts with lower Raman shift. Considering only partial features achieves an accuracy of 92%. Finally, we ensemble the three ResNet strategies by an 8 : 1 : 1 weight, and the final ensemble model attains a 95.8% accuracy for distinguishing CRC samples from normal tissues. Table 3 reports the accuracy, precision, and recall of each model. Figure 5 demonstrates the confusion matrix of the ensemble model performance on the test set.

**Table 3:** Comparison of 1D-ResNet classification performances with three strategies.

Method	Accuracy	Precision	Recall
Resnet_original	0.946	0.939	0.943
Resnet_intensify	0.953	0.943	0.954
Resnet_split	0.920	0.909	0.915
Ensemble	0.985	0.980	0.986

### 3.3. Comparison Study

**Figure 6:**
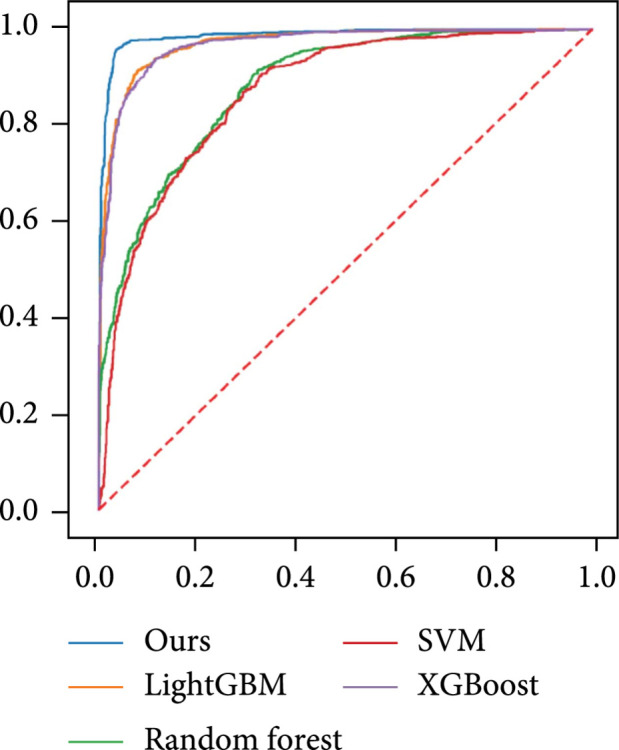
The ROC curves of several machine learning methods for classifying two tissue types in Raman spectra data. Blue curve: our ensemble 1D-ResNet method (AUC=~0.98); orange curve: LightGBM method (AUC=~0.97); green curve: Random Forest method (AUC=~0.88); red curve: SVM method (AUC=~0.87); purple curve: XGBoost method (AUC=~0.96).

As discussed in Section 2.6, we conducted comparison experiments to verify the performance of our 1D-ResNet in distinguishing Raman spectra. As shown in Table 4, we trained several classification models based on SVM, Random Forest, LightBoost, and XGBoost, respectively. The baseline machine learning methods were implemented by scikit-learn package [42]. We selected the hyperparameters with exhaustive grid search and ensured that each algorithm can converge eventually. Each experiment was conducted with random parameter initialization three times, and the best performance was reported. Table 4 shows the results of the comparison experiments, in which the accuracy, AUC, precision, recall, and F1-score of each method are reported. Our proposed ensemble 1D-ResNet method obtains the best classification performance among all the machine learning methods considered. Figure 6 displays the receiver operating characteristic (ROC) curve of each method. SVM and Random Forest show relatively poor performance in AUC (~0.86 and ~0.88). The two boosting methods, LightGBM and XGBoost, attain better AUC (~0.96). Our ensemble 1D-ResNet yields the best AUC (0.986).

**Table 4:** Performance comparison of several machine learning classification methods.

Method	Accuracy	AUC	Precision	Recall	F1-score
SVM	0.7763	0.8679	0.7482	0.7836	0.7655
Random Forest	0.7837	0.8817	0.7388	0.8288	0.7812
LightBoost	0.9148	0.9697	0.9019	0.9170	0.9094
XGBoost	0.9055	0.9674	0.8957	0.9023	0.8990
Ours	0.9850	0.9981	0.9818	0.9859	0.9834

### 3.4. Visualization and Interpretation

**Figure 7:**
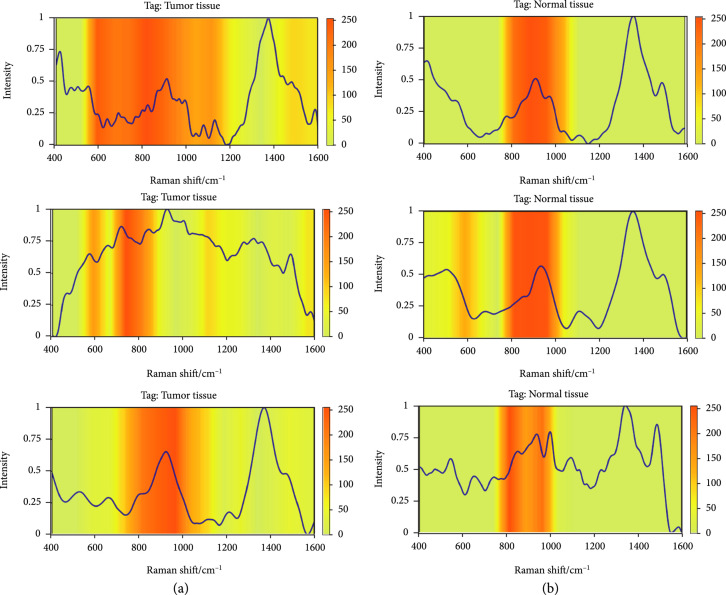
Visualization of activated parts in spectra data using Grad-CAM. (a) is for tumor tissue spectra and (b) is for normal tissue data.

Based on class activation mapping (CAM) analyses, we sought to provide some intuitive insights into the capability of our 1D-ResNet model on Raman spectra data. We collected tumor tissue spectra data as well as normal tissue data. We fed the spectra data to 1D-ResNet and plotted the CAM using Grad-CAM [43]. As shown in Figure 7, although the target data seem similar, the 1D-ResNet model focuses on different Raman shift regions. For the tumor tissue spectra, the model has a wide range from 450cm^-1^ to 1200cm^-1^, while it has a narrower range from 800cm^-1^ to 1000cm^-1^ for the normal tissue spectra. The experimental visualization results can be used to explain the differences in components from the tumor tissue data. Table 5 lists the potential components in different Raman shifts as reported in [44]. This might offer a potential tool to extract more invariant feature representations and recognize components in adverse environments.

**Table 5:** Raman peak assignment.

Raman shift/cm^-1^	Intensity	Vibrational mode	Component
497	Weak	s (S-S)	DNA
599	Mid	v (N-H)	Cytosine
725	Mid	b*v* (N-H)	Adenine
810	Mid	v ($C_{a}$-O-P-O-$C_{b}$)	Phosphodiester
876	Weak	v (O-O)	Lactate
890	Weak	δ (C-O-H)	Glucose
936	Strong	s ($C_{a}-C_{b}$)	Polysaccharides
986	Strong	v ($C_{a}-C_{b}$ or C-O)	Ribose
1014	Mid	v (pyr half-ring)	Glucose
1125	Weak	v ($C_{\beta }-\text{methyl}$)	Protein
1130	Mid	s (C-N)	D-mannose
1156	Weak	v (pyr half-ring)_sym_	Glucose
1210	Weak	bv (N-H), s (C-H)	Amide
1328	Strong	τ(${\text{CH}}_{2}$)	DNA/RNA
1447	Strong	b*v* (C-H)	Collagen

Abbreviations: v: vibration; bv: bending vibration; τ: twist vibration; s: stretching vibration; δ: scissoring vibration.

## 4. Discussions

Our work demonstrated that Raman spectroscopy is a potential technique for the detection of CRC tissues during colorectal cancer surgeries. As a popular spectral technology, Raman spectroscopy is nondestructive to the sample, and it does not need a complex procedure of sample preparation. The Raman spectra can be acquired quickly within seconds. Besides, Raman spectra are sensitive to organic composition changes yet not to water and air. These advantages of Raman spectra make it possibly useful for examining the target tissues directly in surgeries. Before deep learning methods showed their capability to analyze big data, Raman spectra relied on experienced chemists to outline the characteristic peaks. Previous studies are aimed at enhancing the peak intensity through designing and injecting proper nanoparticles into the targets [16–18]. However, such nanoparticles are considered a kind of drug and are hard to apply in clinical surgeries. Some previous work analyzed Raman spectra data with machine learning methods [24–26] and reported over 90% accuracy in classifying tumors and normal tissues, but the data amounts in such studies were limited. For example, in [41], it achieved a 100% SVM classification accuracy with only 20 Raman spectrum data collected from one patient.

In this work, we collected over 20000 Raman spectrum data from 26 CRC patients, covering the most common types of colorectal cancer patients. We developed the 1D-ResNet method in this work with three strategies to enhance the CRC classification performance. The results indicated that our deep learning method is competent for detecting colorectal tumors in Raman spectra data. Since interpreting deep learning algorithm results is always a problem, we used Grad-CAM to visualize the activated parts in Raman spectra data. From the highlighted Raman peak assignment, the corresponding components in tissues can be examined in experiments to verify that our proposed deep learning method can capture correct feature peaks.

## 5. Conclusions

In conclusion, we applied deep learning techniques to detect colorectal cancer via Raman spectra data. An ensemble 1D-ResNet model was proposed that achieves accurate and automatic decoding of Raman spectra-encoded data. This method could address the issues of low efficiency and poor stability of data analyses using traditional machine learning methods. Our 1D-ResNet can accurately and stably classify all the test data with good convergence in our comparison experiments. The decoding performance of 1D-ResNet was far superior to that of common traditional machine learning models. Visualization results highlighted the component differences in colorectal tumor tissues. Our proposed method could enable the applications of Raman spectra in clinical CRC diagnoses. Future work will concentrate on the detection and analyses of actual colorectal tumor samples.

## Data Availability

The preprocessed Raman spectra data used to support the findings of this study are available from the corresponding authors upon request.
